# Biobanking research on oncological residual material: a framework between the rights of the individual and the interest of society

**DOI:** 10.1186/1472-6939-14-17

**Published:** 2013-04-02

**Authors:** Luciana Caenazzo, Pamela Tozzo, Renzo Pegoraro

**Affiliations:** 1 Department of Molecular Medicine, University of Padova, Via Falloppio 50, 35121 Padua, Italy; 2 Fondazione Lanza, Via Dante 55, 35139 Padua, Italy

**Keywords:** Biobanks, Consent, Oncological residual material, Cancer biobanks, Residual materials biobanks, Informed consent, Ethics, Research, Solidarity

## Abstract

**Background:**

The tissue biobanking of specific biological residual materials, which constitutes a useful resource for medical/scientific research, has raised some ethical issues, such as the need to define which kind of consent is applicable for biological residual materials biobanks.

**Discussion:**

Biobank research cannot be conducted without considering arguments for obtaining the donors’ consent: in this paper we discuss to what extent consent in biobank research on oncological residual materials has to be required, and what type of consent would be appropriate in this context, considering the ethical principles of donation, solidarity, protection of the donors’ rights and the requirements of scientific progress. Regarding the relationship between informed consent and tissue collection, storage and research, we have focused on two possible choices related to the treatment of data and samples in the biobank: irreversible and reversible anonymization of the samples, distinguishing between biobank research on residual materials for which obtaining consent is necessary and justified, and biobank research for which it is not. The procedures involve different approaches and possible solutions that we will seek to define. The consent for clinical research reported in the Helsinki Declaration regards research involving human beings and for this reason it is subordinate to specific and detailed information on the research projects.

**Summary:**

An important ethical aspect in regard to the role of Biobanks is encouraging sample donation. For donors, seeing human samples being kept rather than discarded, and seeing them become useful for research highlights the importance of the human body and improves the attitude towards donation. This process might also facilitate the giving of informed consent more willingly, and with greater trust.

## Background

The tissue biobanking of biological residual materials constitutes a good resource for bio-medical research and has raised some ethical issues, such as the need to understand the beliefs and values of the donor patients [1], including the motivations underlying consent or refusal to give the samples to biobanks [2], and the balance between the protection of individual and society’s common good.

Biological residual materials are obtained from pathological waste from surgical activities (surgical removal and biopsies of neoplasias); they are collected for analysis and are usually stored in formalin in Pathology Departments.

The conservation of paraffin blocks for decades certainly represents an enormous resource as a biobank, even though its utility is somewhat limited for molecular analyses, considering that many studies have demonstrated that prolonged fixation in formaldehyde entails cross-linking reactions and above all DNA denaturation, with consequent partial or total failure of the polymerase chain reaction (PCR) amplification process, compromising the quality of amplified DNA [3-7].

When the collected material is no longer necessary for diagnostic purposes, it can be stored with the purpose of future genetic studies: in this case samples should be frozen immediately after collection in order to maintain a good quality of DNA/RNA or, more generally, of macromolecules. Various investigations have examined the integrity of macromolecules within periods of time after the excision of the tissue sample. The non-conclusive nature of the published studies, and the impossibility of precisely defining the susceptibility of various genes and their expression to hypoxic damage, highlight that the collection of tissues destined for storage in biobanks should take place as quickly as possible [8-12].

For these reasons, the choice of sampling neoplasias that are destined for research is guided by the diagnostic needs of the tissue sample and the timing and mode of sample conservation, from excision to transport to the Pathology laboratory, storage in the laboratory and the disposal of the tissues.

During the operation it is necessary to send the representative part of the neoplasia to the pathologist for analysis. The representative fragment of the tumor has to be sent to the pathologist for diagnosis, but the surgeon may also keep other fragments of tissue not appropriate for diagnostic procedure, so the surgeon can decide immediately to keep part of the tissue and store it. The pathologist fixes the tissue in formalin and encloses in paraffin a fragment that is representative of the tumor. From the paraffin blocks he/she obtains the histological slides to analyze under the microscope and makes the diagnosis. Once the amount of tissue necessary for immediate or future diagnosis has been preserved, the pathologist can store the residual materials in formalin solution, or store them in small pieces at −80°C.

Biobanks of frozen oncological residual materials are undoubtedly expensive; however, it could be useful and important to consider their possible involvement in particular oncological studies, such as linking abnormalities in the tissue to disease aetiology, and analyzing gene expression and gene regulation to identify the mechanisms of tumorigenesis and to study epigenetic phenomena [13].

For these reasons, a reorganization of the “tissue conservation” in Pathology Departments would also be useful for fresh samples, and would require new organizational strategies, from the presence of a pathologist in the operating theatre to sampling techniques. Moreover, the evermore frequent need to determine molecular alterations on tissues collected years ago makes standardization of all tissue management procedures fundamental, starting with fixation (the process of fixation constitutes the first and essential step for the tissue conservation for pathological purposes).

Moving on from these technical explanations to how residual materials can be obtained, regarding the relationship between informed consent and tissue collection, and storage and research, in this paper we highlight two possible choices related to the treatment of data and samples in the biobank: irreversible and reversible anonymization of the samples. The procedures involve different approaches and possible policy making strategies that we will seek to define, considering that donation of residual biological material, collected for diagnosis, could be beneficial for society from the perspective of the common good, rather than being directly affecting the individual’s health status.

## Discussion

### Informed consent in oncological residual material biobanks

In general, the ethical discussion on biobank research involves a number of questions concerning the relationship between the individual, society and biobanks.

In general, researchers obtained and used tissue samples for defined purposes which were disclosed to contributors. In these cases the consent form regarded the specific research project. Today, in the case of biobanks, the donor of the sample may not be affected by the research, and future uses of the samples may be unknown at the moment of the consent. However, biobank research cannot be conducted without considering arguments for obtaining the donors’ consent [14,15].

While the consent proposed by the Helsinki Declaration [16] is specific for clinical trials, which involve humans beings and are clearly oriented towards pharmacological or other interventional studies, which have to be described exactly, in biobank research the consent for residual material involves research whose future peculiarity and aims cannot be clearly identified at moment of consent.

As suggested by Gefenas et al. [17] it seems important to discuss justifiable divergences from the paradigm proposed by the Helsinki Declaration about consent. It seems appropriate “to tune different levels of regulatory stringency (including regulations on acceptable types of consent) to different types of biological materials according to the interests of the donors involved”.

The question is to what extent consent in biobank research on oncological residual materials has to be required and what type of consent would be appropriate in this context [17-19], considering the influence coming from the ethical aspects of donation, solidarity and the respect of donor’s autonomy through the informed consent, considering, as the most important aspect, how to find a balance between the individual’s rights and the interest/benefit of society from the perspective of the common good.

Particular attention is given by the current literature to different form of applicable consent [17,20-23] in the field of biobanking.

We may consider and justify the action of leaving the residual neoplastic sample in the biobank as a “donation” that constitutes an advantage for medical research in general. Such a donation could be considered beneficial for society in the perspective of the common good in biobanking, an aspect that we will further discuss.

In particular, in the case of oncology many factors may play a central role in the decision-making process of donation. In fact, depending on the type of cancer and the stage of the disease, different physical and mental symptoms can be noted as pain, anxiety, and depression related to the risk of serious side-effects or death; furthermore the genetic information related to cancer may have effects on the family as a whole community and on its single members.

In biobank research, the distinction between the *anonymization* and *pseudonymization* of samples and data, as a matter of personal data protection, has obtained a great attention from an ethical point of view [24]. When data and samples are anonymized, according to the European understanding, the link between the original donor and the data or material is deleted. When they are pseudonymized, the donor can always be re-identified by the biobank operators using a code. Researchers who work with the biobank samples do not receive the full identity of the original sample but just its code. It is sometimes argued that in the strict sense of the word (i.e., absolute anonymity) can never be achieved, because theoretically, genetic samples and data can be re-attributed to the donor. Art 3 of Appendix to the Recommendation Rec (2006)4 of the Committee of Ministers of Europe to member states on research on biological materials of human origin [25] states that anonymity is already achieved when a person cannot be identified “with reasonable efforts”. This can be interpreted as a “pragmatic” definition of anonymity which should not be confused with absolute anonymity.

Regarding the relationship between informed consent and tissue collection, storage and research, we have focused on two possible choices related to the treatment of data and samples in the biobank: irreversible and reversible anonymization (the latter meaning the previous cited *pseudonymization*) of the samples, meaning, on the basis of what is established in the Recommendation we have cited, that “Identifiable biological materials are those biological materials which, alone or in combination with associated data, allow the identification of the persons concerned either directly or through the use of a code” as a reversible anonymization and, “Non-identifiable biological materials, hereafter referred to as “unlinked anonymized materials”, are those biological materials which, alone or in combination with associated data, do not allow, with reasonable efforts, the identification of the persons concerned” as irreversible anonymization.

We refer in this context to the term “reversible anonymization” instead of pseudonymization to highlight the contrast with the completely different process of “irreversible anonymization”.

This procedure is applicable only to the samples to be collected in the future and not to those already in storage.

Consent options could be performed following an OPT-OUT model which consists in informing the patient before the surgical operation about the possibility to donate completely anonymous tissue samples obtained from residual oncological materials, which would otherwise be discarded after the operation. Alternatively, the OPT-IN model is represented by a classic request for consent before the operation; this can be assimilated to a broad and precautionary consent, meaning that in the consent form provided for research use of residual material the specifics of the future research projects are unknown [17].

The procedures involve different approaches and possible solutions that we will seek to define.

### Irreversible anonymization of the sample

The main question regards which kind of informed consent is necessary for a procedure of irreversible anonymization of samples and data.

If the tissue sample will be irreversibly anonymized and no information regarding the donor can ever be obtained from it, the donor is guaranteed in his/her privacy but does not have any return of results because it will be impossible to link the data to the donor’s sample.

In this case, we think it would be best to consider a “presumed consent”, rather than an actual consent request.

In case of the OPT-OUT model a special hospital staff would be in charge of the information process by direct (or individual) information or by other general strategies, for example in the information for the consent to the surgery procedure.

In other words, the practical form of the OPT-OUT consent can be simply explained as follows: “I collect your residual material sample with a standardized procedure, then I inform you that it will be stored and preserved and you may refuse. If you say “no” to its preservation, this is a refusal and I will discard the sample; otherwise, if you don’t reply I consider you in agreement, allowing the preservation and use of the anonymized sample”.

Irreversible anonymization should be considered an exceptional procedure because with this kind of samples it is not possible to trace any information about disease history, lifestyle, personal and health information. However, in our opinion, this may be useful when the alternative is the loss of the sample, when the donor would have refused to give his residual material with an OPT-IN form, and when it could be useful to collect samples for basic research in which the above mentioned information on the donor is not necessary.

In this particular situation it should be a duty to renovate the informed consent process for an OPT-OUT choice.

Irreversibly anonymized samples can constitute a resource for basic research where information about the donor is not necessary for permitting the saving of samples linked to individual information for disease-oriented research. However, this option should not be not offered simultaneously as alternative to the OPT-IN form, but just as a second choice, because the general goal of a biobank is to obtain samples related to disease history, lifestyle, personal and health information, so the OPT-IN model is overriding.

### Reversible anonimyzation of the sample

In this case, we think that information and consent should be clear and in a “OPT-IN” form, because the donor’s sample is identified by a code that constitutes the link with the donor’s data.

The decision to donate residual tissues for biobanking may take little from the donor in terms of time commitment or harm above and beyond the donation of oncological residual materials from tissues collected for diagnostic or therapeutic purposes. On the other hand, the potential benefits to society are relatively unclear, given the open-ended nature of residual material banking research, which may make less clear to the donors the reasons why they should participate.

Therefore, we should think of a broad consent with the following suggestions on the information:

for how long the samples will be stored;

who has access to donor’s information (name, surname, qualification, degree, etc.) and who is the person responsible for the link when, for the reversible process, a code is attributed to the sample;

what the sample will be used for: it is important to indicate the specific branch of research, in the view of obtaining consent from the beginning for conducting, at a later time, analyses without the necessity of requesting further consent, (for example a broad consent may be requested for research into the genetics of colon cancer or regarding tumor etiopathology of the colon);

the value of the donation for medical research.

In general we have to consider that often, in these situations, patients must discover what residual material biobanking is, why it is beneficial and how they may contribute when they meet the doctor before a surgical operation. Decisions to donate tissues are therefore likely to be strongly influenced by the information that clinicians and researchers provide about the risks and benefits of biobanking [18].

The consent processes for residual material banking should take account of the range of potential motives that may influence a patient’s decision to donate tissues [26]. Such a process might involve asking the patients what they know about this kind of biobank, what they expect from donating their residual tissues for research, whether they have any anxieties about donating their residual materials, and if so, what they are and how they came to their decision to donate. Tissue donation appears to be viewed as part of a generalized or indirect form of reciprocity in which the good will of others is reciprocated by the donor’s contribution and by which the act of donation contributes to society’s common good.

In particular, in case of oncological research the decision of donating may be influenced on one hand by an attitude of altruism that influences in a positive way the patient’s decision process in favor of donation; on the other hand, feelings of fear, anger or refusal related to the conditions of the illness may lead the patient to deny any kind of contribution to the common good. Furthermore, genetic information concerning cancer may provide information about a relative and may cause concerns for family members who are already involved in managing the disruptive effects of a cancer diagnosis.

The decision-making process and the information process will take time, but ultimately it is likely to increase the validity and importance of the consent process and increase engagement with biomedical research and trust in its importance.

We can assume that in both donations, made with an OPT-OUT consent for irreversible and OPT-IN for reversible sample anonymization, the assessment whether to keep the biological residual materials as a donation would be made in a perspective of solidarity, and cancer patients should be helped in perceiving it as a possibility to contribute to the common good, instead of refusing any social exchange with other people.

The difference in the form of consent is justified, in our opinion, by the fact that irreversible anonymization implies a lower risk (in general terms) for the donor, and with the purpose of usefulness of a biobank for the whole society rather than for the single patient, a presumed silent assent seems to be enough and to facilitate the process of donation.

Since the sample is irreversibly anonymized, the OPT-OUT is justified by a research objective and there is no violation of individual rights because there is no risk of the violation of privacy, and the autonomy of the donor is respected.

In contrast, the reversible anonymization of the sample could represent a greater risk for the donors, regarding, for example, the violation of privacy, as well as the harm from group stigmatization [27,28], cultural or religious objection to particular forms of research [26], the return of results, and the donation should be explicitly intended and therefore should derive from an explicit consent. An explicit consent is necessary, since in this situation the OPT-OUT consent is an overly weak expression for consent for donors. Biobank research produces risks different from other types of medical research, for example pharmacological or interventional clinical trials, because the risk of bodily harm is very small or even non-existent. The risks regarding biobank research could be: the knowledge/communication of a specific disease entity (for example, in case of mental/infectious diseases), the stigmatization of individuals, or the misuse of scientific health data for other purposes. Furthermore, incidental findings cannot be excluded.

Regarding the context of donation we can cite the work by Morrell et al. in New South Wales (Australia) based on a series of qualitative interviews with patients who had donated tissue to a tumor bank [29]. The study shows that “participants in this study overwhelmingly expressed their willingness to donate leftover tumor tissue for research because of “the good” that it might bring to others… these “others” included family, friends, disease community members (present and future), future generations and at times simply “anyone”.

In general, in the case of tissue donation to a biobank in which donors spontaneously expressed concern that their donations should benefit others, they did not perceive that they would be assuming a cost through the act of donation.

The donation of tissue residual materials to a biobank is made, altruistically, with the purpose to improve the scientific research and promote people’s health. People donating tissue residual material know that this kind of donation will be of no direct benefit for their health status or in medical terms. Researchers have no contact with donors and the donors may be easily changed and chosen randomly in the population. The duty to contribute to medical research considered here is not free from restrictions for researchers, especially in case of cancer patients, who are in need of special care and multidimensional support. These restrictions are provided, under appropriate circumstances, by ethical committees and by the law with the aim to guarantee trustworthiness and confidence in research.

Another important ethical aspect regarding biobanks on oncological residual material is to promote an educational social plan encouraging sample donation.

As remarked by Hoedemaekers et al. “For the adequate development of basic rights and shared values something else is essential: participatory self-rule. Every individual should be able to engage in public debate, and actively take part in social and community life to foster and promote the important values and liberties. This means that social participation is also seen as a core value” [30,31].

Regarding biobanking this duty doesn’t seem to be hard, if we consider that for donors, seeing human samples being kept rather than discarded, and seeing them becoming useful for research, since donation might benefit others and contribute to a generalized system of solidarity, the importance of the human body is highlighted and the attitude towards donation is improved.

This process might also facilitate the giving of informed consent more trustfully and willingly, granting, in this context, that donation permission is nothing more than an automatic response.

Concerning this aspect, in Morrell’s study it is reported that donors had a high level of willingness to donate tumor tissue for research. Donation was often considered to be “not a big deal” given that it involved the use of tissues which would be discarded anyway and was perceived as involving little or no credible risk [29].

Despite the sense that tumor donation was “a lot of fuss about nothing,” donors expressed considerable satisfaction at the thought that their donation might benefit others and contribute to a generalized system of reciprocity.

The expression of OPT-OUT consent will be a condition for the irreversible anonymization of data and samples, this form of “consent” doesn’t involve risks for donors and constitutes an advantage for society’s common good; and the silent assent consent seems to be sufficient to facilitate the process of a direct donation.

In contrast, the reversible for example anonymization of the sample could represent a greater vulnerability for the donors, regarding the violation of privacy and the return of results and the donation should be explicitly intended and therefore should derive from an explicit consent.

The structure and content of the informed consent for biobank should be different from the one reported by the Helsinki Declaration regarding clinical trials, which doesn’t seem to fit adequately to the purposes of biobanking, considering the necessity of adjusting the existing notions of informed consent and government regulation to the new realities of donors’ samples donation to biobanks [32].

The consent for clinical research reported in the Helsinki Declaration regards research involving human beings and for this reason it is subordinate to specific and detailed information on the research projects. The OPT-IN consent for oncological residual material biobanks, which involves research on reversible anonymized samples, should be viewed as a “broad” consent, well-structured in terms of information, where the approval of future research projects, different from the original ones, has to be the responsibility of the Ethical Committee. In this way we can guarantee the balance between the protection of individual rights and the interest/benefit of society in creating and maintaining biobanks. Biobank research, aiming to improve medical and scientific knowledge obviously related to prevention, treatment and therapy of diseases that is clearly in the interest of the single donor, is considered as belonging to the society as a whole. This will mean that when patients are faced with decisions of whether or not to participate in residual materials biobanking, they will be better equipped with a motivational understanding of its significance, which is necessary to reflect upon and interpret the situation and what is being asked of them, and to realize their active solidarity for society’s common good.

## Summary

Biological residual materials can be obtained from surgical activities or from pathological waste material collected for analysis and stored in formalin. This material can be stored in Biobanks with the purpose of future research. Regarding the relationship between informed consent and tissue collection, storage and research, two choices are possible: irreversible or reversible sample anonymization. Informed consent places itself as a balance between the expression of the individual rights and society’s interest. Given the importance of the information process on tissue residual material biobanking to the decision-making process about donation, we argue that issues surrounding residual material collections for biobanking should enter into public debate.

## Competing interests

The authors declare that they have no competing interests.

## Authors’ contributions

LC initiated the idea of the paper. LC and PT contributed to the drafting and RP to the subsequent revision of the paper. All authors read and approved the final manuscript.

## Pre-publication history

The pre-publication history for this paper can be accessed here:

http://www.biomedcentral.com/1472-6939/14/17/prepub
